# Polymorphic variants of the dopamine receptor gene DRD2 (rs6277, rs1800497) in adolescents with problematic video game use

**DOI:** 10.18699/vjgb-24-74

**Published:** 2024-10

**Authors:** S.Yu. Tereshchenko, K.V. Afonicheva, I.V. Marchenko, M.V. Shubina, M.V. Smolnikova

**Affiliations:** Scientific Research Institute of Medical Problems of the North – a separate division of the Federal Research Center “Krasnoyarsk Science Center” of the Siberian Branch of the Russian Academy of Sciences, Krasnoyarsk, Russia; Scientific Research Institute of Medical Problems of the North – a separate division of the Federal Research Center “Krasnoyarsk Science Center” of the Siberian Branch of the Russian Academy of Sciences, Krasnoyarsk, Russia; Scientific Research Institute of Medical Problems of the North – a separate division of the Federal Research Center “Krasnoyarsk Science Center” of the Siberian Branch of the Russian Academy of Sciences, Krasnoyarsk, Russia; Scientific Research Institute of Medical Problems of the North – a separate division of the Federal Research Center “Krasnoyarsk Science Center” of the Siberian Branch of the Russian Academy of Sciences, Krasnoyarsk, Russia; Scientific Research Institute of Medical Problems of the North – a separate division of the Federal Research Center “Krasnoyarsk Science Center” of the Siberian Branch of the Russian Academy of Sciences, Krasnoyarsk, Russia

**Keywords:** gene polymorphism, dopamine, teenagers, problematic video game use, game addiction, Internet addiction, полиморфизм генов, дофамин, подростки, проблемное использование компьютерных видеоигр, игровая зависимость, интернет-зависимость

## Abstract

Problematic video games use, as a specific form of problematic Internet use, is widespread among adolescents and can have negative effects on their mental and somatic well-being. An increasing incidence of addictive video gaming, as well as the overuse of the Internet, among the young population makes the current study of susceptibility factors, including the genetic component, relevant. There has been a number of investigations related to the involvement of gene variants of the neurotransmitter system in the development of Internet addiction, with the results being different for various ethnic groups. The dopamine type 2 receptor gene (DRD2) is one of the candidate genes for susceptibility to video game addiction. The aim of the work was to study polymorphic variants of the dopamine receptor gene DRD2 (rs6277, rs1800497) in Russian adolescents with problematic use of computer video games. A sampling of 407 adolescents aged 14.1 ± 1.8 years was tested, of which 56 (13.8 %) were identified as having problems with the pathological use of video games use based on the GASA scale results. Boys in the sample proved to be addicted to video games more than girls (p = 0.041). As a result of comparing the allele frequency of DRD2 (rs6277), a tendency to a higher frequency of the minor allele T was revealed in the group of adolescents with problematic video game use compared with adolescents without problematic video game use (i. e. 0.563 and 0.466, respectively, p = 0.06). When using the dominant inheritance model, it was revealed that adolescents with problematic use of video games were statistically significantly more likely to carry the T (CT+TT) allele (p = 0.04, OR = 2.14, CI = 1.01–4.53). The T allele DRD2 (rs6277) is associated with low expression of the dopamine receptor D2 and leads to decreasing the density and affinity of extrastriatal dopamine type 2 receptors, which is associated with impaired social communication as well. We suggest that the presence of CT and TT genotypes of rs6277 DRD2 may be a potential risk factor for developing problematic video game use in adolescents.

## Abstract (ru)

Проблемное использование видеоигр как специфическая форма проблемного использования Интернета широко распространено среди подростков и может оказывать негативный эффект на их психическое и соматическое благополучие. Рост зависимости от пользования видеоиграми, как и Интернетом, среди молодого населения делает актуальным изучение факторов подверженности к ним, в том числе генетической составляющей. Существует ряд исследований, посвященных изучению вовлеченности полиморфных вариантов генов системы нейромедиаторов в развитие Интернет-зависимости, результаты которых различаются в разных этнических группах. Ген рецептора дофамина второго типа DRD2 является одним из кандидатных генов подверженности к патологической зависимости от использования видеоигр. Целью работы было исследование полиморфных вариантов гена рецептора дофамина DRD2 (rs6277, rs1800497) у русских подростков с проблемным использованием компьютерных видеоигр. Протестирована выборка из 407 подростков в возрасте 14.1 ± 1.8 года, у 56 (13.8 %) из которых на основании результатов оценки шкалы GASA было выявлено проблемное использование видеоигр. Мальчики в выборке чаще были зависимы от видеоигр, чем девочки (p = 0.041). В результате сравнения частоты аллелей DRD2 rs6277 обнаружена тенденция к большей частоте минорного аллеля T в группе подростков с проблемным использованием видеоигр по сравнению с подростками без проблемного использования видеоигр (0.563 и 0.466 соответственно, p = 0.06). В доминантной модели наследования у подростков с проблемным использованием видеоигр статистически значимо чаще встречалось носительство аллеля T (СТ+ТТ) (p = 0.04, OR 2.14, CI = 1.01–4.53). Носительство аллеля T DRD2 rs6277 ассоциировано с низкой экспрессией дофаминового рецептора D2 и приводит к снижению плотности и аффинности экстрастриарных дофаминовых рецепторов второго типа, что сопряжено в том числе с нарушением социальной коммуникации. Мы полагаем, что наличие генотипов CT и TT rs6277 гена DRD2 может выступать потенциальным фактором риска развития проблемного использования видеоигр у подростков.

## Introduction

Problematic video game use among adolescents is a pressing challenge in modern society and is characterized by excessive passion for video games, leading to negative consequences in various areas of life: social, educational, somatic and psychological (Griffiths et al., 2012; Paulus et al., 2018; Männikkö et al., 2020).

Since video games are currently associated with high Internet use in the vast majority of cases, the problematic use of video games is considered by most experts to be a specific problematic use of the Internet, or Internet addiction. Several synonymous terms can be found in the literature available, essentially describing a single psychological construct: game addiction (ICD-11), Internet gaming disorder (DSM-5), gaming disorder, pathological video gaming, excessive video game use, compulsive gaming, problematic digital gaming, problematic online gaming, problematic video game use (PVGU). These are the terms that are often used interchangeably in scientific publications, however, there may have some semantic aspects depending on the context and the theoretical background of the study. The European Research Group recommends using the term “Problematic Use of the Internet” for generalized Internet addiction and its particular types, i. e. “Problematic Social Media Use” and PVGU (Fineberg et al., 2022). Only one of the many specific types of addictive Internet behavior, namely PVGU, is currently considered to be a mental disorder (Internet Gaming Disorder, DSM-5; American Psychiatric Association, 2013; Gaming Disorder, ICD-11, 2019).

As shown in a systematic review by S. Mihara and S. Higuchi (Mihara, Higuchi, 2017), the prevalence of PVGU varies from 0.7 to 27.5 % and, like generalized Internet addiction, is highly dependent on the questionnaires used and addiction assessment criteria. As with generalized Internet addiction, the prevalence of PVGU shows higher prevalence values in Asian countries with predominantly Mongoloid population compared to other regions (Sussman et al., 2018).

Very few studies have been devoted to finding the genetic basis of Internet addiction as opposed to other types of addictions (e. g. substance abuse or gambling). For example, the first twin study based on an examination of 825 children aged 10–12 years in the Chinese population was conducted in 2014, with the authors being able to estimate the proportion of total variability due to genetic effects, which varied from 58 to 66 % depending on gender (Li M. et al., 2014). Similar results were obtained a little later in the study of Turkish (19–86 %) (Deryakulu, Ursavaş, 2014), Dutch (48 %) (Vink et al., 2016), Australian (41 %) (Long et al., 2016) and German (21–44 %) (Hahn et al., 2017) twin cohorts. Although these data are limited by the sample size and different ethno-geographic conditions, there is likely to be a tendency towards a greater contribution of genetic factors in males. Thus, the presence of a genetic component in developing Internet addiction has been convincingly demonstrated by twin studies using various populations as an example, however, to date, specific genes involved in the mechanisms of such heritability have not been precisely identified.

Therefore, candidate genes are in active study, their polymorphic variants can disrupt the functioning of neurotransmitter systems and cause mental and behavioral disorders. One of them is the dopamine receptor gene DRD2 (Kim et al., 2022). Dopamine is a hormone responsible for motivation, desire and addiction, functionally associated with the “pleasure centers”. Dopaminergic brain neurons form the nigrostriatal, mesolimbic, mesocortical, tuberoinfundibular pathways (Kolotilova et al., 2014). The D2 receptor, classified as inhibitory, is present in high concentrations in the striatum, olfactory tubercle, amygdala, nucleus accumbens, hypothalamus, substantia nigra and ventral tegmental area (Ford, 2014; Arnsten et al., 2015). The human dopamine receptor gene DRD2 is located on chromosome 11 (q22-q23) and is polymorphic, with different genetic variants altering the availability and expression of the dopamine D2 receptor gene, which affects receptor sensitivity and density (Magistrelli et al., 2021). The rs6277 polymorphism in exon 7 of the DRD2 gene is a substitution of the amino acid serine for cysteine (Ser311Cys). The homozygous CC genotype of rs6277 DRD2 causes low sensitivity to dopamine in the striatum (Hänninen et al., 2006). However, outside the striatum (extrastriate area), this genotype has a high affinity to dopamine D2 receptors (Liu et al., 2014; Smith et al., 2017; Della Torre et al., 2018). The dopamine binding potential by D2 receptors in the striatum is higher in carriers of the TT genotype of rs6277 DRD2, while the opposite effect is observed in the extrastriate area (Hänninen et al., 2006). A decrease in DRD2 density in the striatum and environmental effect are known to result in the development of addictions, including alcohol, drugs, computer games (Hill et al., 2008; Bhaskar, Kumar, 2014; Gao et al., 2017; Anokhin et al., 2019; Picci et al., 2022). However, according to the published data, it is debatable which allele (C or T) of rs6277 DRD2 is associated with addiction to psychoactive substances (Hill et al., 2013). The T allele of rs6277 DRD2 is shown in some studies to be associated with an increased tendency to pathological addiction to video games (Kim et al., 2022).

However, it is worth noting that genetic factors represent only one aspect of the tendency to addictive behavior, and the influence of the environment and sociocultural factors also play an essential role. Thus, it is known that a stressful environment combined with the T allele of rs6277 DRD2 causes a decrease in the ability to control craving for computer games (Kim et al., 2022). Individuals with the homozygous TT genotype of rs6277 DRD2 have been shown to respond better to nicotine replacement therapy than carriers of the C allele (Hill et al., 2008). The C allele variant of rs6277 DRD2 causes a hypodopaminergic state manifesting as a reduced ability to suppress responses to reward-related stimuli (Machulska et al., 2016; Richter et al., 2017; Rył et al., 2024). Carriers of the homozygous CC genotype of rs6722 DRD2, who were abused or experienced traumatic life events in childhood, have been demonstrated to have a high degree of impulsivity and more frequent alcohol consumption in adulthood (Klaus et al., 2021). It is reported that the risk of developing such addiction is higher in adult C allele carriers of rs6277 DRD2, whereas in adolescents (11–13 years old), this allelic variant may be protective against the development of dependence on psychoactive substances, as well as predispose to a later onset of alcohol consumption (Picci et al., 2022).

The rs1800497 polymorphism of the DRD2 gene causes an amino acid substitution of glycine for lysine (Glu713Lys), which leads to a specificity change of dopamine receptor binding. According to some data, this polymorphism is called DRD2/ANKK1 Taq1A because it is located within the protein kinase PKK2 gene (Ankyrin Repeat and Kinase Domain Containing 1 – ANKK1), a protein of the post-receptor intracellular signal transmission system (Gafarov et al., 2019). The rs1800497 DRD2 polymorphism is also frequently studied in the context of neuropsychiatric disorders and addictions (Volkow et al., 1996; Pohjalainen et al., 1998). The A1 allele (T) carriers were found to have a 30 % decrease in the density of dopamine D2 receptors in the brain striatum, resulting in poor attention and learning ability, an increase in anxiety, and an association with “reward deficiency” and “novelty seeking” syndrome (Klein et al., 2007; Kushnarev, 2022). The presence of the minor T allele of rs1800497 was similarly shown in the work (Pohjalainen et al., 1998) to be associated with a reduced number of dopamine binding sites in the brain. It has been suggested that there is an association between the A1/A1 (TT) and A1/A2 (TC) genotypes of rs1800497 of the DRD2 gene with “reward deficiency” syndrome (Klein et al., 2007). “Reward deficiency” syndrome causes various mental and behavioral disorders, i. e. nicotine and drug addiction, gambling addiction, ADHD, autism spectrum disorders, eating disorders with compulsive overeating (Pohjalainen et al., 1998). It was found that male carriers of the allelic T variant of rs1800497 are more likely to suffer from addiction to online games (Paik et al., 2017). This allelic variant is also more common in people addicted to playing video games to satisfy their seeking of reward (Werling, Grünblatt, 2022). Thus, people with a low number of dopamine D2 receptors tend to search for extreme ways to enjoy life. Impaired sensitivity of dopamine receptors causes a decrease in people’s ability to draw the right conclusions from negative experiences, since dopamine is involved in learning processes and provides the opportunity to effectively learn from mistakes.

The aim of this study was to investigate polymorphic variants of the dopamine receptor gene DRD2 (rs6277, rs1800497) in adolescents with problematic video game use for possible associations between genetic variants and behavioral aspects of gaming addiction to be identified.

## Materials and methods

In the present study, psychological and genetic testing of 407 adolescents aged 12–18 years was carried out. All adolescents involved in the study were Russians (verified by both mother and father nationality). Informed consent was obtained from the adolescents or their parents (legal representatives), followed by notification of the voluntary and confidential nature of the study. The study participants were asked to fill out a demographic data questionnaire (gender, age, nationality of mother and father), and a translated version of the Game Addiction Scale for Adolescents (GASA) questionnaire (Lemmens et al., 2009). The GASA questionnaire includes seven questions concerning behavioral disorders in adolescents caused by overuse of Internet games. Each question is assessed on a 5-point scale: “never” (0 points), “rarely” (1 point), “sometimes” (2 points), “often” (3 points), “very often” (4 points). According to the criteria proposed by the authors of the questionnaire (Lemmens et al., 2009), having PVGU was determined (if the teenager answered any four or more of seven questions – “sometimes”, “often” or “very often”).

After completing the questionnaire, adolescents were asked to provide saliva samples in special containers. Saliva samples were collected using the “Saliva DNA Collection and Preservation Devices” (Cat. No. RU 49080, Norgen Biotek Corp., Canada). DNA was isolated from saliva samples using the DIAtom DNA Prep kit (Isogene Lab, Russia). Genotyping of polymorphic variants rs6277 and rs1800497 DRD2 was performed using TaqMan technology with probes and primers (DNA Synthesis, Russia) and a reaction mixture (Syntol, Russia) on a Rotor-Gene 6000 device (Qiagen, Germany). The study was approved by the Ethics Committee of FRC KSC SB RAS (Protocol No. 12 dated 12.18.2018).

Statistical analysis was performed using Statistica v.10 software (StatSoft Inc., USA). Differences in categorical data were evaluated using Pearson’s χ2 test with Yates’s correction, and the differences in quantitative data were evaluated with Student’s t-test.

## Results

Descriptive statistics of the main variables are presented in Table 1. The mean age of the 407 tested adolescents was 14.1 ± 1.8 years, the ratio of boys/girls = 174 (42.8 %)/233 (57.2 %). PVGU was detected in 56 adolescents (13.8 %) based on the GASA scale assessment results (Table 1). Boys had significantly higher mean scores on the game addiction scale than girls. In addition, more PVGU adolescents were detected among boys compared to girls.

**Table 1.**
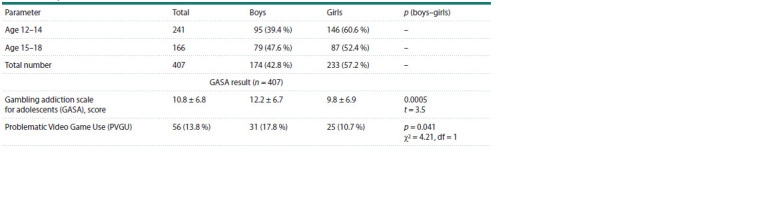
Descriptive statistics of main variables Notе. Data are presented as n (%) and mean ± standard deviation

The genotype distribution frequency of the polymorphic variants of the DRD2 gene (rs6277 and rs1800497) in the adolescents studied corresponds to their distribution in Caucasian populations (according to the website ensembl.org). The distribution of genotype frequencies was consistent with the Hardy–Weinberg equilibrium, both for PVGU cases and for the group without PVGU. Thus, the allele frequencies of the selected polymorphic variants in the study population were balanced and, therefore, applicable to association studies

The distribution of genotype and allele frequencies of the rs6277 and rs1800497 polymorphisms of the DRD2 gene depending on the PVGU presence and absence is given in Tables 2 and 3, respectively. The genotype frequency distribution of polymorphic variants of rs6277 (DRD2) did not differ significantly between the PVGU group and in the group without PVGU ( p = 0.12) (Table 2). At the same time, when comparing the frequencies of alleles of DRD2 rs6277, a clear trend towards a higher frequency of the minor T allele in the group of adolescents with PVGU was found compared to the group without PVGU ( p = 0.06). Analysis of polymorphic variants of rs1800497 of the DRD2 gene showed no significant differences in the frequencies of genotypes and alleles between the groups with and without PVGU (Table 3).

**Table 2.**
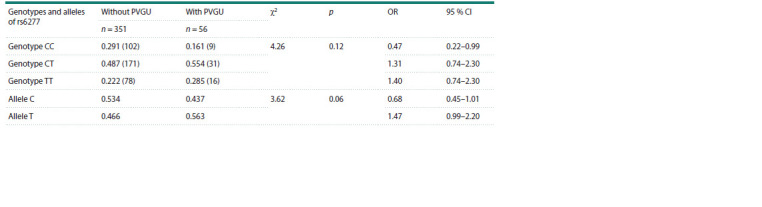
Distribution of genotype and allele frequencies of rs6277 for the DRD2 gene in adolescents with and without PVGU

**Table 3.**
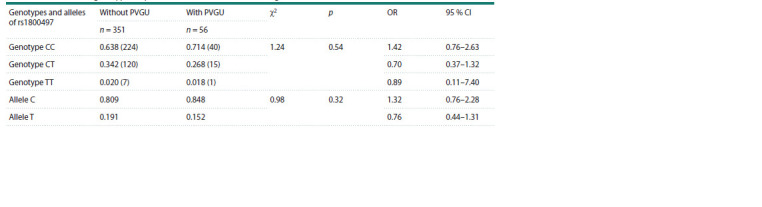
Distribution of genotype frequencies of rs1800497 for the DRD2 gene in adolescents with and without PVGU

Then, we analyzed the distribution of genotype frequencies of the rs6277 polymorphism of the DRD2 gene using the dominant model of inheritance, where heterozygotes and homozygotes for the minor allele of rs6277 of the DRD2 gene (CT and TT, respectively) were combined (Table 4).

**Table 4.**
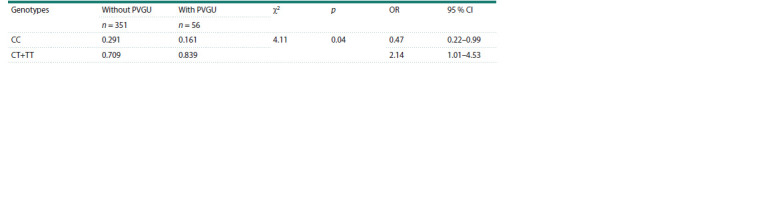
Distribution of genotype frequencies of the rs6277 polymorphism of the DRD2 gene in adolescents with and without PVGU

According to the obtained results, in the group of adolescents with PVGU, carriage of the T allele (genotype CT+TT) was statistically significantly more common compared to adolescents without PVGU. Calculation of the odds ratio (OR) demonstrated a significant association between carriage of the T allele and the presence of PVGU in adolescents.

## Discussion

The overall PVGU frequency in the studied sample of Russian adolescents was 13.8 %, which is not significantly different from our previously obtained data on the prevalence of computer game addiction, resulting from a large-scale epidemiological project (n = 4,514, PVGU prevalence – 10.4 %) (Tereshchenko et al., 2022). Boys in the sample of the present study were more often addicted to video games than girls (p = 0.041), which is consistent with the data of the mentioned project and the results of other epidemiological studies using the GASA questionnaire (Mihara, Higuchi, 2017; Tereshchenko et al., 2022). The genotypes and alleles distribution in the studied sample is similar to their frequency in the global population of European descent according to the 1000 Genomes Project and HapMap databases (website: ensemble.org), both for rs6277 and rs1800497. Thus, in terms of the prevalence of the main variables, the population studied is typical enough, and the findings can be successfully extrapolated to other adolescent of European populations

We have found that the CT and TT genotype carriers of the rs6277 polymorphism of the DRD2 gene, that is, the T allele carriers, according to the results obtained using the dominant model of inheritance, exhibit signs of PVGU significantly more often than adolescents with the CC genotype.

The T allele carriers of rs6277 of the DRD2 gene are known to have a lower density and affinity for dopamine D2 receptors in all brain regions (including the prefrontal cortex), excluding the striatum, compared to carriers of the C allele – C/C > C/T > T/T (Hirvonen et al., 2009; Smith et al., 2017). Low DRD2 density in extrastriatal brain region can lead to certain psychophysiological consequences. In particular, the functional effects of the availability of these receptors in extrastriatal regions, including the cortex and thalamus, have been considered in the study devoted to the role of extrastriatal DRD2 (Takahashi et al., 2006). The review includes postmortem examinations as well as in vivo studies in humans and animals, considering the role of low functional activity of extrastriatal DRD2 for schizophrenia (Takahashi et al., 2006). Low availability of D2/3 receptors in extrastriatal regions in adult males with socio-communicative deficits in autism has been indicated by C. Murayama et al. to be associated with reduced dopamine receptor density (Murayama et al., 2022).

The T allele carriers of rs6277 of the DRD2 gene were shown to be less active in suppressing impulsive tendencies to undesirable actions than the C allele carriers (Colzato et al., 2010). In the study by O.H. Della Torre et al., it was found that the T allele carriers of rs6277 of the DRD2 gene (6–18 years old) were characterized by problems with impulse control, self-control of emotions and volitional personality change (Della Torre et al., 2018). As a theoretical model confirming the genetic data, the authors of the study cite the opinion of G.S. Dichter et al. that a decrease of the dopaminergic activity is associated with learning problems and a lack of selfdiscipline (Dichter et al., 2012).

Our data on the association between the T allele carriage of rs6277 of the DRD2 gene and PVGU in adolescents correspond with the study results of E. Kim et al. directly relating the PVGU severity and the T allele carriage in college students (b = 19.58, p = 0.04) using regression analysis (Kim et al., 2022). Two other studies conducted on samples of adults didn’t show such an association (Paik et al., 2017; Rył et al., 2024). The inconsistency of the results obtained can be explained by the differences in age, gender, ethnicity, and number of sampling size of the aforementioned studies. In particular, the influence of the genetic component on addictions may be manifested differently in adolescents and adults. Adolescence is characterized by different time trajectories in developing the limbic system and prefrontal cortex (Casey et al., 2008). Delayed development of the prefrontal cortex compared to the limbic system during adolescence results in weakened cortical inhibition on underlying subcortical structures and increased impulsivity, which contributes to a high risk of developing addictive behavior (He, Crews, 2007).

We believe that the association between the T allele carriage of rs6277 DRD2 and PVGU in adolescents and students, which Kim et al. (Kim et al., 2022) and our research team have found, provides the theoretical and empirical background. Carrying the T allele of rs6277 leads to a decrease in the density and affinity of extrastriatal dopamine D2 receptors (Hirvonen et al., 2009; Smith et al., 2017) and a peculiar phenomenon of “dopamine desensitization”, which is associated with a reduced sensitivity to reward, increased impulsivity, lack of self-discipline (Colzato et al., 2010; Della Torre et al., 2018; Weinstein, Lejoyeux, 2020; Kim et al., 2022), as well as a possible impairment of social communication (Takahashi et al., 2006; Murayama et al., 2022).

Hyporeactivity of the orbitofrontal cortex and decreased dopaminergic function in this brain region are associated with hyposensitivity of the reward system, promoting transgressive behavior, delinquency, and substance abuse (Matthys et al., 2013). Certain DRD2 variants were suggested to possibly contribute to the development of a hypodopaminergic state, with partial availability of dopamine receptors determining reduced sensitivity to reward (Alcaro et al., 2021). The latter may lead to the adolescent aiming to receive additional stimulation of the dopaminergic system, which manifests as addictive behavior including an active persistent reward component, such as compulsive use of video games (Weinstein, Lejoyeux, 2020; Kim et al., 2022) or gambling.

Increased impulsivity and impairment of social connection turn out to be the most important predictors of the development of generalized Internet addiction and its specific form – i. e. PVGU. Impulsivity and self-control are associated with a wide range of behavioral characteristics. Empirical studies have shown that people with high self-control are better at controlling their thoughts, regulating their emotions and suppressing their impulses than individuals with low self-control (de Ridder et al., 2012). Low self-control and high impulsivity are closely related to delinquency, crime, antisocial behavior, externalizing behavior, victimization and addictive disorders. One of the psychiatric disorders most associated with Internet addiction has been known to be Attention Deficient Hyperactivity Disorder, characterized by high behavioral impulsivity (Wang et al., 2017). A large number of psychological studies have shown that Internet-addicted behavior is closely associated with low self-control/high impulsivity (Li W. et al., 2016; Li S. et al., 2021; Yu et al., 2021). A meta-analysis of 40 neurophysiological studies of problematic Internet use have shown that, regardless of content, Internet-addicted behavior is characterized by significant impairment in inhibitory control, decision-making and working memory (Ioannidis et al., 2019). A meta-analysis by M. Zhang et al. has demonstrated a common pattern of structural brain changes in chemical and behavioral addictions, i. e. changes in the prefrontal and insular cortex, associated with increased impulsivity (Zhang et al., 2021).

A rare T allele of the rs1800497 DRD2 polymorphism is also associated with low expression of the dopamine D2 receptor gene in the prefrontal cortex and has been found by S.-H. Paik et al. to be more common among Korean men (19–47 years old) with Internet gaming addiction (Paik et al., 2017). This variant is also more common in Korean young adults (high school students and college students) with PVGU and high reward dependence (Han et al., 2007). However, our study results did not provide any statistically significant differences between different genotypes and alleles of the rs1800497 polymorphism of the DRD2 gene in groups with and without signs of PVGU. The inconsistency in the analysis results may be due to the ethnic and gender characteristics of the samples, as well as the use of different psychometric tools to verify PVGU. In particular, there are pronounced ethnic differences in the genotype and allele frequencies of the rs1800497 DRD2 polymorphism in representatives of Caucasian and Mongoloid populations that may have a great impact.

## Conclusion

The research results of polymorphic variants of the dopamine receptor gene DRD2 in adolescents with PVGU allow one to conclude that genetic factors are important for developing this behavioral disorder. The availability of CT and TT genotypes for the polymorphic locus rs6277 of the DRD2 gene may be a potential risk prediction of developing PGVU in adolescents. Further study of the genetic basis of behavioral disorders will provide personalized approach to the prevention and treatment of game addiction, taking into account the patient’s genetic profile.

## Conflict of interest

The authors declare no conflict of interest.
